# Immunohistochemical detection of ERβ in breast cancer: towards more detailed receptor profiling?

**DOI:** 10.1054/bjoc.2001.1721

**Published:** 2001-04

**Authors:** G P Skliris, P J Carder, M R J Lansdown, V Speirs

**Affiliations:** Molecular Medicine Unit, University of Leeds, St James's University Hospital, Clinical Sciences Building, Leeds, LS9 7TF; Pathology Department; Breast Unit, St. James's University Hospital, Leeds, LS9 7TF

**Keywords:** ERβ, breast cancer, immunohistochemistry, formalin-fixed, paraffin-embedded material

## Abstract

Oestrogen receptor (ER) is used routinely to predict endocrine responsiveness in patients with breast cancer. A second ER, ERβ has been described but its significance remains undefined; most studies have described mRNA levels rather than protein expression. Here, we demonstrate for the first time, immunohistochemical detection of ERβ in archival breast tumours. © 2001 Cancer Research Campaignhttp://www.bjcancer.com


*British Journal of Cancer* (2001) **84**(8), 1095–1098
© 2001 Cancer Research Campaign
doi: 10.1054/ bjoc.2001.1721, available online at http://www.idealibrary.com on IDEAL®

### Immunohistochemical detection of ERβ in breast cancer: towards more detailed receptor profiling?

**GP Skliris[1], PJ Carder[2], MRJ Lansdown[3] and V Speirs[1]**

[1]Molecular Medicine Unit, University of Leeds, Clinical Sciences Building, St James's University Hospital, Leeds LS9 7TF; [2]Pathology Department and [3]Breast Unit, St. James's University Hospital, Leeds, LS9 7TF

**Summary** Oestrogen receptor (ER) is used routinely to predict endocrine responsiveness in patients with breast cancer. A second ER, ERβ has been described but its significance remains undefined; most studies have described mRNA levels rather than protein expression. Here, we demonstrate for the first time, immunohistochemical detection of ERβ in archival breast tumours. © 2001 Cancer Research Campaign http://www.bjcancer.com

**Keywords**: ERβ; breast cancer; immunohistochemistry; formalin-fixed; paraffin-embedded material

2 human ER isoforms exist, the 'classic' ERα (Green et al, 1986) and the more recently identified ERβ (Kuiper et al, 1996; Mosselman et al, 1997). Immunohistochemical analysis of ERα in breast cancer is now routine practice and plays a major role in the selection of adjuvant therapy in patients with this disease (Harvey et al, 1999). Pilot work at the mRNA level suggests a role for ERβ in tamoxifen resistance (Speirs et al, 1999a). However, few studies have investigated protein expression in archival material. This is fundamental because there is no guarantee that mRNA will be translated into functional protein. Recently, immunohistochemical detection of ERβ was reported in frozen sections of normal (Speirs et al, 2000) and malignant breast (Jarvinen et al, 2000). However, for ERβ to be of clinical use it is essential to identify a suitable antibody for its detection in formalin-fixed, paraffin-embedded tumours, since the majority of clinical samples are processed in this way. Therefore, we have optimized an antigen retrieval histochemical technique using a suitable antibody, capable of detecting ERβ protein in archival human breast carcinomas.

### MATERIALS AND METHODS

With ethical approval, 65 breast carcinomas (35 infiltrating ductal, 15 lobular, 9 tubular/cribriform, 4 mucinous, 1 DCIS, 1 medullary) and 8 normal breast tissues were randomly selected. None of the patients had been treated pre-operatively with endocrine therapy. Detection of ERβ by immunohistochemistry (IHC) was performed using a monoclonal antibody (ERβ-14C8, Abcam, Cambridge, UK). The antibody was affinity-purified and raised by immunizing mice with a recombinant protein encoding 1–153 amino acids of human ERβ sequence. According to the manufacturers 14C8 did not cross react with hERα. This was further confirmed by incubating 14C8 and anti-ERα (1D5, Dako, UK) with an ERβ blocking peptide (sc-6820P; Autogen Bioclear, UK). Appropriate positive controls (normal human breast) and negative (omission of primary antibody, incubation with blocking peptide) were also included.

*Received 30 November 2000*
*Revised 24 January 2001*
*Accepted 24 January 2001*

*Correspondence to:* V Speirs

5 μm sections were mounted on Superfrost slides (BDH, Poole, UK), microwaved in 0.01 M citrate buffer, pH 6.0, for 27 min at 480 W then incubated overnight with 5 μg $ml^{-1}$ primary antibody at 4˚C. Next, sections were incubated with biotinylated secondary antibody followed by streptavidin/peroxidase complex (Vectastain Quick Kit) at room temperature and visualized with 3,3′-diaminobenzidine (all Vector, Peterborough, UK). Sections were counterstained with haematoxylin, dehydrated and coverslipped. Slides were observed under a Nikon light microscope and images captured using Lucia software (version 4.51). A simple scoring system was used involving assessment of both staining intensity and percentage positivity, generating a numerical score of 0–8. A score of >2 was classified as positive (Allred et al, 1998). Staining was scored independently by two authors (GPS, PJC). Statistical analysis was performed using Fisher's exact test.

### RESULTS

Consistent, strong ERβ staining was detected specifically in cell nuclei of both epithelial and myoepithelial cells from normal breast ducts and lobules, both in breast reduction specimens and normal tissue adjacent to tumours (Figure 1A, B). 74% of carcinomas (48/65) exhibited specific nuclear staining for ERβ (Figure 1C,D,E). Light cytoplasmic staining was visualized in some tumours, scored as ERβ negative if seen alone, whilst occasional weak to moderate staining was seen in surrounding stromal cells. Specific staining was abolished where primary antibody was substituted with blocking serum or pre-absorbed with an ERβ blocking peptide (Figure 1F). No effect of this peptide was seen with primary antibody directed against ERα (data not shown). Results obtained between different test runs were consistently reproducible.

Compared with infiltrating ductal carcinomas, invasive lobular, tubular/cribriform and mucinous tumours showed significantly increased ERβ positivity ($P = 0.02$, Table 1), illustrating the differences in biological characteristics between distinct tumour types. However, when the results were correlated with clinicopathological features, ERβ was significantly associated with ERα, PR and well-differentiated tumours (Table 1).

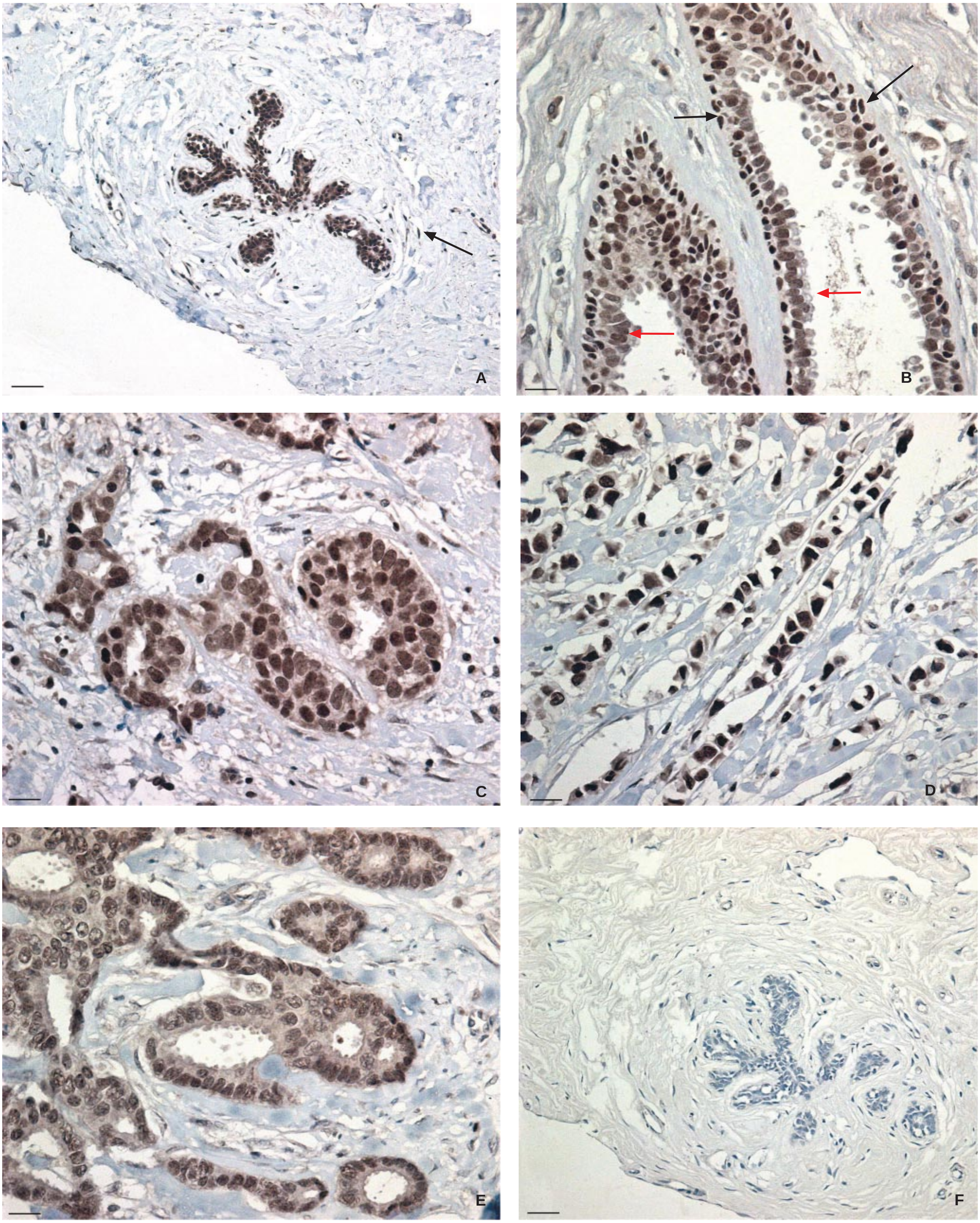

**Figure 1** (**A**) Immunohistochemical detection of ERβ protein in cell nuclei of breast ducts. Note occasional positivity in stromal cells (arrow). (**B**) ERβ expression in the nuclei of both epithelial (red arrows) and myoepithelial cells (black arrows) of normal mammary glands. (**C**) Invasive ductal carcinoma showing specific nuclear staining for ERβ. (**D**) Strong ERβ immunoreactivity localized in cell nuclei of an invasive lobular carcinoma. (**E**) Invasive tubular/cribriform tumour expressing ERβ protein. (**F**) Serial section of (**A**) showing abolition of specific staining following pre-absorption of primary antibody with an ERβ blocking peptide. Bars = 1 μm

 *© 2001 Cancer Research Campaign*

**Table 1** Association of ERβ with clinico-pathological features in 65 breast carcinomas

| Parameter | ERβ+ | ERβ– | *P* value |
|---|---|---|---|
| Receptors[a] | | | |
| ERα+ | 40 | 10 | 0.03 |
| ERα– | 6 | 7 | |
| PR+ | 31 | 6 | 0.002 |
| PR– | 8 | 11 | |
| ERα+/PR+ | 32 | 5 | ERα+/PR+ vs. ERα+/PR– = 0.024 |
| ERα+/PR– | 3 | 4 | |
| ERα–-/PR+ | 0 | 0 | |
| ERα–/PR– | 5 | 7 | ERα+/PR+ vs. ERα–/PR–= 0.004 |
| Lymph node[a] | | | |
| + | 15 | 5 | 0.77 |
| – | 29 | 13 | |
| Menopause | | | |
| Post- | 44 | 13 | 0.19 |
| Pre- | 4 | 4 | |
| Tumour type[b] | | | |
| Ductal | 22 | 13 | Ductal vs. lobular = 0.18 |
| Lobular | 13 | 2 | Ductal vs. all other types = 0.02 |
| Tubular/cribriform | 8 | 1 | Ductal vs. tubular/cribriform = 0.23 |
| Mucinous | 4 | 0 | |
| Tumour size | | | |
| ≤ 2 cm | 31 | 12 | 0.77 |
| > 2 cm | 17 | 5 | |
| Histological grade | | | |
| I | 15 | 3 | I vs. II = 1.0 |
| II | 24 | 4 | I vs. III = 0.04 |
| III | 9 | 10 | II vs. III = 0.009 |

[a]Unknown ERα status = 2, unknown PR status = 9, unknown node status = 3. [b]Excludes one medullary carcinoma and one DCIS.

### DISCUSSION

Our results unequivocally demonstrate that ERβ can be routinely detected, in archival, formalin-fixed, paraffin-embedded breast tumours using the 14C8 monoclonal antibody. This may have profound clinical implications, as it will now allow detailed receptor analysis in the routine diagnostic setting.

ERβ was co-expressed with ERα in 74% of tumours and showed a strong association with PR and well-differentiated tumours. This is in concordance with Jarvinen (2000), but refutes the observations of Dotzlaw et al (1999). However, the latter study was conducted at the mRNA level, highlighting that caution should be observed when extrapolating mRNA results to those of protein.

Many ERβ antibodies have become commercially available over the last year. Despite this, there is a paucity of studies investigating this protein in breast tumours. Taylor and Al-Azzawi (2000) reported ERβ in a range of formalin-fixed normal human material, including breast. They used 2 polyclonal antibodies raised against the N- and C-termini of hERβ (06–629, Upstate Biotechnology; PAI-310, Affinity Bioreagents, USA respectively). In addition, Jarvinen et al (2000) reported successful detection of ERβ in frozen tumours, using a different polyclonal antibody (PAI-313, Affinity Bioreagents, USA), but interestingly their attempts with paraffin material were unsuccessful. Frozen sections are performed infrequently in routine practice, so there is a need for a suitable antibody and a reliable technique for use in paraffin sections. To our knowledge, this is one of the first studies reporting ERβ in paraffin-embedded human breast carcinomas. The availability of 14C8 should help resolve conflicting reports proposing ERβ as a good (Jarvinen et al, 2000) or poor (Dotzlaw et al, 1999; Speirs et al, 1999a,b) prognostic factor in breast cancer.

Presence of ERβ in breast tumours may help explain the differential tissue- or gene-specific effects, which have been reported with oestrogens/antioestrogens (Paech et al, 1997). The relative expression of ER subtypes seems to alter during tumorigenesis in terms of mRNA (Leygue et al, 1998); if this is borne out at protein level, it could have relevance with respect to novel selective ER modulators, currently being developed against specific ER phenotypes. When these become available, they could offer the possibility of individually tailored therapy based on the particular receptor profiles of breast carcinomas.

### ACKNOWLEDGEMENTS

We thank Yorkshire Cancer Research and Northern and Yorkshire NHS Executive for financial support. Thanks to Jane Ramsdale for technical assistance.

### REFERENCES

Allred DC, Harvey MJ, Berardo M and Clark GM (1998) Prognostic and predictive factors in breast cancer by immunohistochemical analysis. *Mod Pathol* **2**: 155–168

Dotzlaw H, Leygue E, Watson PH and Murphy LC (1999) Estrogen receptor β messenger RNA expression in human breast tumour biopsies: Relationship to steroid receptor status and regulation by progestins. *Cancer Res* **59**: 529–532

Green S, Walter P, Kumar V, Krust A, Bornert JM, Argos P and Chambon P (1986) Human oestrogen receptor cDNA: sequence, expression and homology to v-*erb*-A. *Nature* **320**: 134–139

*© 2001 Cancer Research Campaign*

Harvey JM, Clark GM, Osborne CK and Allred DC (1999) Estrogen receptor status by immunohistochemistry is superior to the ligand-binding assay for predicting response to adjuvant endocrine therapy in breast cancer. *J Clin Oncol* **17**: 1474–1481

Jarvinen TAH, Pelto-Huikko M, Holli K and Isola J (2000) Estrogen receptor β is co-expressed with ERα and PR and associated with nodal status, grade and proliferation in breast cancer. *Am J Pathol* **156**: 29–35

Kuiper GG, Enmark E, Pelto-Huikko M, Nilsson S and Gustaffson JA (1996) Cloning of a novel receptor expressed in rat prostate and ovary. *Proc Natl Acad Sci USA* **93**: 5925–5930

Leygue E, Dotslaw H, Watson PH and Murphy LC (1998) Altered estrogen receptor α and β messenger RNA expression during human breast tumorigenesis. *Cancer Res* **58**: 3197–3201

Mosselman S, Polman J and Dijkema R (1996) ER-β: identification and characterisation of a novel human estrogen receptor. *FEBS Lett* **392**: 49–53

Paech K, Webb P, Kuiper GGJM, Nilsson S, Gustaffson J-A, Kushner PJ and Scanlan TS (1997) Differential ligand activation of estrogen receptors ERα and ERβ at AP-1 sites. *Science* **277**: 1508–1510

Speirs V, Malone C, Walton DS, Kerin MJ and Atkin SL (1999a) Increased expression of estrogen receptor β mRNA in tamoxifen-resistant breast cancer patients. *Cancer Res* **59**: 5421–5424

Speirs V, Parkes AT, Kerin MJ, Walton DS, Carleton PJ, Fox JN and Atkin SL (1999b) Co-expression of estrogen receptor-α and -β: Poor prognostic factors in human breast cancer? *Cancer Res* **59**: 525–528

Speirs V, Adams IP, Walton DS and Atkin SL (2000) Identification of wild type and exon 5 deletion variants of estrogen receptor β in normal human mammary gland. *J Clin Endo Metab* **85**: 1601–1605

Taylor AH and Al-Azzawi F (2000) Immunolocalisation of oestrogen receptor beta in human tissues. *J Molec Endocrinol* **24**: 145–155

 *© 2001 Cancer Research Campaign*
